# Supplementary material for: Machine learning to predict mesenchymal stem cell efficacy for cartilage repair
Source: PLoS Comput Biol. 2020 Oct 7;16(10):e1008275. doi: 10.1371/journal.pcbi.1008275 (PMC7571701; doi:10.1371/journal.pcbi.1008275)
Supplement: S1 Text — (PDF) [file pcbi.1008275.s001.pdf]

Supporting information

In this supporting information, we provide additional details and validate the hyperparameters for our machine learning method. In Algorithm A, we outline the algorithm to calculate the uncertainty of our neural network. It is an iterative cycle until the result is converged. In Figure A we measure the model accuracy against other methods and also explore the hyperparameter selection. In Figure A(i) we compare to other machine learning algorithms, Neural Network (NN), Random Forest (RF), K-Nearest Neighbor (KNN), Collective Matrix Factorization (CMF), and Multiple Linear Regression (MLR). For each method, we also perform individual investigation on the selection of input properties and optimize its hyperparameters to deliver maximum accuracy and measure through the same leave-one-out cross-validation test as in the paper. Note the CMF algorithm has an internal component selection parameter so we did not manually select the input properties for it. For RF, KNN, and MLR that require all inputs to be present, we either use imputation algorithm to fill in the database or perform machine learning using only complete rows of data, and also measure accuracy using machine learning model trained on now complete data. We measure the performance through the R^2 and we see in Figure A(i) that the NN delivers the best accuracy, followed by RF, and then the other methods. Furthermore, the predictive power of the RF is improved using our imputation algorithm for the missing data. Therefore, we use neural networks with our imputation methods.

Having now decided that we are using neural network, we now address the hyperparameter selection for it. First, in Figure A(ii) we compare using different activation function. We see that the tanh function delivered the best R^2, closely followed by the sigmoid function, but they can by distinguished within their uncertainties. These are similar shape functions and it is not a surprise that they give similar performance, however the relu function is significantly worse at accuracy, this is unsurprising as the relu function had a gradient discontinuity, which is rarely seen in real-life biological systems. Therefore, we adopt the tanh function. Next, in Figure A(iii) we choose the learning rate: too low a learning rate never progresses, and too high a learning rate causes instability and never converges. We choose the learning rate of 0.05 for the stable performance and efficiency. Then, in Figure A(iv) we test the number of hidden nodes with blue curve represents the cross-validation R^2. Too few hidden nodes are unable to capture the behavior while too many hidden nodes overfits the training data. Therefore, we choose 6 hidden nodes for our neural network.

In Figure B, we perform a computational experiment to validate the effective of the imputation method by treating some known values as missing. The original database has 18% data missing, which we now remove some data points randomly and make it more sparse, then calculate the R^2 value. The blue dots represents R^2 values obtained from our neural network with different missing data percentages, the shaded area represents the uncertainty at each percentage and the purple line is the fitted average result. The R^2 value decreased to 0 with large missing data percentage when the model is unable to explain the response but to predict the average of the data. This experiment further confirms the validation of our imputation methods.

There is open access to the data and codes at `https://doi.org/10.17863/CAM.52036`.

Algorithm A A ensemble model to measure the ANN's prediction uncertainty.

---

initialize the training data (N points);
while *the number of training models is not reached* **do**
  draw N points with replacement at random;
  train the neural network on the data;
  record the model;
end
Result: Evaluate the performance of predictions, such as averages and standard deviations, from all different models.

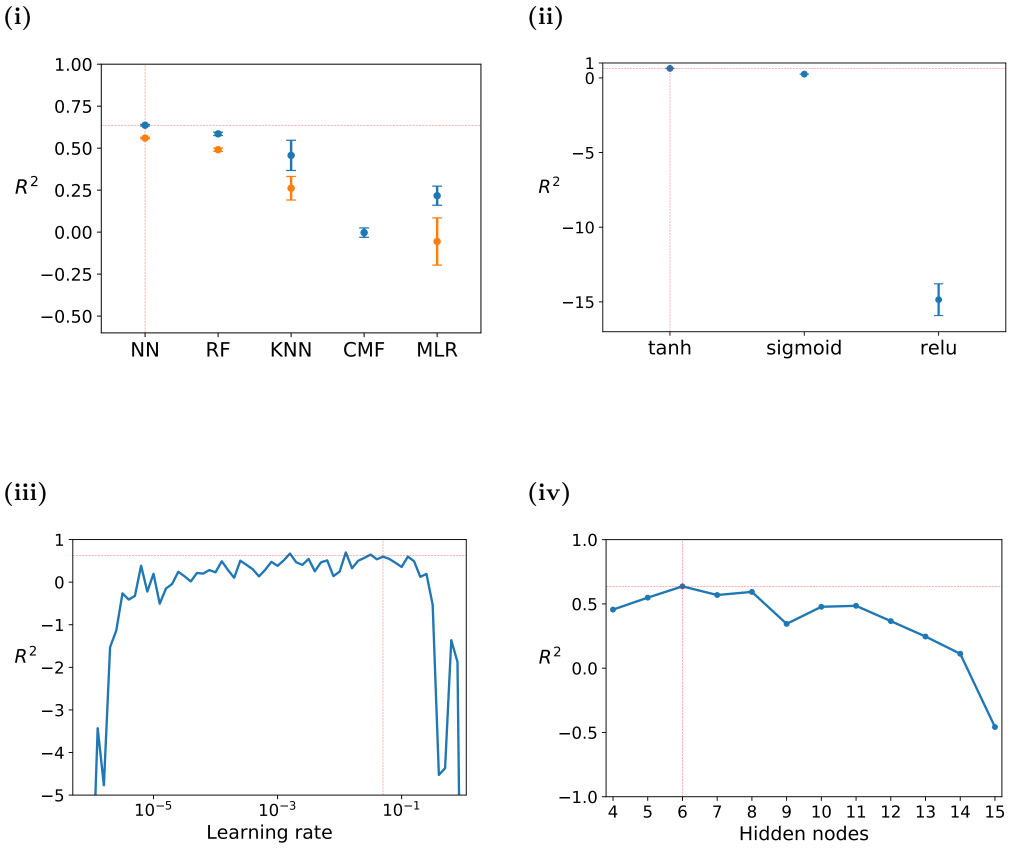

Figure A The model performance across different algorithms and hyperparameters. (i) Comparison of the performances of several algorithms, Neural network (NN), Random Forest (RF), K-Nearest Neighbor (KNN), Collective Matrix Factorization (CMF) and Multiple Linear Regression (MLR) using our database. The yellow dots are the methods without imputing the underlying missing data in the training dataset, and the blue dots are machine learning methods using the training dataset that has missing data imputed by our bespoke algorithm outlined in Fig 2. The error bars are estimated using the algorithm shown in Algorithm A. (ii) Activation functions, tanh, sigmoid and relu within our neural network. (iii) Determining the optimal learning rate for the neural network. (iv) Determining the optimal number of hidden nodes for the neural network. (The optimal hyperparameter selected is shown by the red vertical line.

The red horizontal line is the R^2 value achieved by our neural network.)

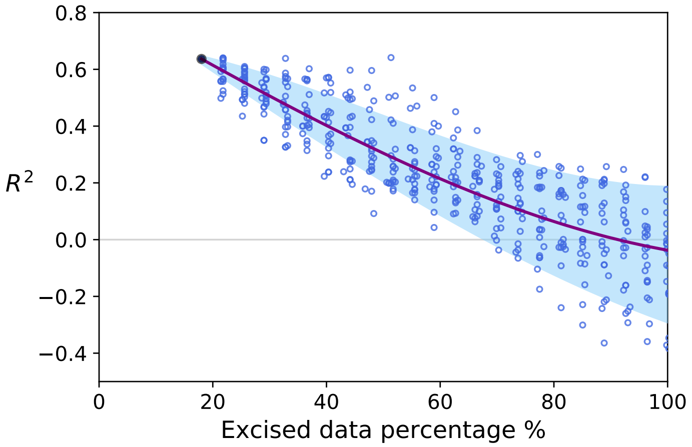

Figure B Performance vs missing data percentage in the database. The original database has 18% data missing (the left black point of the figure). The blue points shows the R^2 value when we excised the original database with higher percentage of missing data. The purple line is the trend line fitted to the average of the data points with the same percentage of missing data. The blue shaded area illustrates the uncertainty of the average R^2 value at each missing data percentage.
