# Supplementary material for: Machine learning to predict mesenchymal stem cell efficacy for cartilage repair
Source: PLoS Comput Biol. 2020 Oct 7;16(10):e1008275. doi: 10.1371/journal.pcbi.1008275 (PMC7571701; doi:10.1371/journal.pcbi.1008275)
Supplement: Attachment — Submitted filename: Response letter.pdf [file pcbi.1008275.s002.pdf]

Manuscript No.: PCOMPBIOL-D-20-00130

Title: Machine learning mesenchymal stem cell efficacy for cartilage repair

Dear Editor,

We thank the Reviewers for their conscientious review of the manuscript and helpful comments. We have used their feedback to improve the manuscript and responded to their comments in full below. We hope that following these changes the manuscript is now ready for publication in the PLOS Computational Biology.

Yours faithfully,

Yu Yang Fredrik Liu, Dr Yin Lu, Professor Steve Oh, and Dr Gareth Conduit

Response to First Reviewer

We are grateful to the Reviewer for taking the time to carefully review our manuscript and providing us with constructive feedback. We are pleased to hear that the Reviewer agrees with us that the manuscript addresses the important biological problem of cartilage repair prediction, and contributes a novel machine learning method to the field. We address the Reviewer's comments in full below:

Reviewer comment

It leaves me the impression that some necessary technical details are omitted in the current m.s., which should be included in the Methods part, or provided as Supplementary Information.

Our response

We thank the Reviewer for raising the concern about technical details. We have now updated the Methods section to make it more clear and added a Supplementary Information section at the end of the manuscript.

Changes to the manuscript from line 108

The ANN has to be trained on a provided data set. The parameters $\{A_{ihj}, B_{hj}, C_{hj}, D_j\}$ are initialized with random values, and varied following a random walk. The new values are accepted, if the new function f(x) models the fixed-point equation f(x) ≡ x better, which is quantitatively measured by the error function,

$$\sigma = \sqrt{\frac{1}{N}\sum_{x\in X}\sum_{j=1}^{I}\left[f_j(x)-x_j\right]^2}\ .$$

This form is also known as the root-mean-square error (RMSE) cost function. The optimization is equivalent to minimization of the RMSE cost function and a steepest descent approach is used.

Reviewer comment

1) The authors claimed uncertainty-quantification and missing data imputation are the unique features of their proposed method. Unfortunately, I am not able to check such claim after reading the Methods part of the m.s., since they are currently presented in a highly abstract and rough way, and the details are not provided even in the Supplementary. For example, in line 103-109, to describe the uncertainty-quantification procedure, they wrote,

“Several separate networks were trained on the data with different weights, and their variance was a measure of uncertainty in the predictions which accounts for both experimental uncertainty in the underlying data and the uncertainty in the extrapolation of the training data [49,50]. This concept is similar when estimating the uncertainty in ensemble models, with the underlying model being changed to neural networks and the uncertainty estimates generated accurately represent the observed errors in the prediction.”

How can different network be obtained by solving a simple minimization problem? How are the weights determined? How many networks are actually trained in author’s data and what about the robustness? Can certain equations/mathematical notations be used to replace such narrative sentences?

Our response

For the simple minimization problem, we can still train different networks or models, and each one is based on different data. The procedure is illustrated in the supplementary material section S1 Algorithm, where we initialize the training data, then draw data points with replacements at random to train a model, and the procedure can be repeated to create different models. Those models’ average has been treated as overall prediction and their standard deviation has been treated as the uncertainty.

The weights are determined by minimizing the root-mean-square error (RMSE). We have now included the equations for the error function at Eq. 2. A hundred networks are used to train the data.

The robustness of the network corresponds to the uncertainty of the network, if the uncertainty is small, then it should be robust. We have shown the histogram of the network’s prediction in Fig 4, where the *x*-axis is the prediction value from the machine learning model minus the *i*-th data point and divided by the uncertainty of that prediction. That number on the *x*-axis should

have an absolute value of 1 with mean value 0, which is what we expect for most data to be one standard deviation away. The results have been overlaid with a standard distribution (red dotted line) and agreed with each other.

Another plot to illustrate the robustness is the accuracy vs the percentage of data validated in Fig 5. If we impute all of the predicted data (use all data in the database), we get the $R^2 = 0.637$ quoted in the paper. If we predict half of the data and reject the other half with large uncertainties, we get the $R^2 = 0.721$.

Changes to the manuscript from lines 115.

In order to measure the uncertainty in the ANN's prediction, we train a number of models simultaneously, and treat their average as the overall prediction and their standard deviation as the uncertainty. The pseudocode is shown in S1 Algorithm, at least 100 training models were used to evaluate the uncertainty. This concept is similar when estimating the uncertainty in ensemble models, with the underlying model being changed to neural networks and the uncertainty generated accounts for both experimental uncertainty in the underlying data and the uncertainty in the extrapolation of the training data.

We have added S1 Algorithm under the Supplementary Information.

Reviewer comment

in line 103-109, when describing the imputation method, they wrote

“We treated all properties as both inputs and outputs of the model and adapt an expectation-maximization algorithm to exploit the relationship across the properties using an iterative approach. ”

What is the objective function of EM algorithm in current setting? Does the iteration finally converge in real-data implementation? Can the iteration procedure be explicitly written in pseudo-code or mathematical expressions?

Therefore, I recommend the authors to revise the writing of methods part and associated SI by adding more details about implementation procedures, making it possible for readers to directly inspect the rationale/plausibility of their methods from the methodology perspective.

Our response

We apologize for not providing sufficiently detailed methodology in the original manuscript. We have extended this section following the Reviewer’s suggestions. This includes details of the iteration cycles, the softerning applied when taking parameters from one cycle to the next, and a flow chart. The text also now clarifies that in our real-life implementation that the iteration cycles were converged.

Changes to the manuscript from line 135

We treated all properties as both inputs and outputs of the model and adapt an expectation-maximization algorithm. This is an iterative approach, where we first provide an estimate for the missing data and use the neural network to iteratively improve the initial estimate.

The algorithm is shown in Fig. 2.

For any unknown properties we first set missing values to the average of the values present in the data set. With estimates for all values of the neural network we then recursively apply the following equation until convergence:

$$x^{n+1} = \gamma x^n + (1 - \gamma)\, x^n \,,$$

where n denotes the iteration step, x^n is a prediction for x obtained from the neural network. The converged result is then returned instead of $f(x^n)$. The function f remains fixed on each iteration of the cycle. A softening parameter, $\gamma \in [0, 1]$, is used to combine the results with the existing predictions, and a $\gamma > 0$ serves to prevent oscillations and divergences of the predictions. Typically, we set γ as 0.5.

Changes to the manuscript in Figure 2

A flow chart (Fig 2.) has been added to illustrate the iteration procedure.

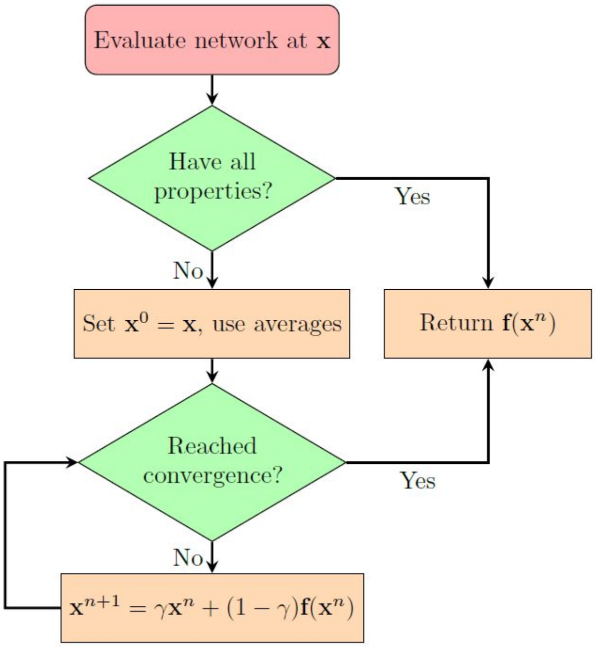

Figure 2

Reviewer comment

2) Implementation details of neural network & reproducibility issue

The m.s. reported the R-square regarding the neural network performance, while seems to neglect reporting other key parameters. I suggest the authors to disclose other parameters and perhaps discuss robustness in Supplementary material (such as learning rate, robustness for hidden layer number and layer width, robustness for activation function (ReLU v.s.tanh) and trained weights), which is generally vital to performance of machine learning techniques. I also think it would be very helpful to make the training codes publicly available for reproducible check and further benchmarking/ application, which is quite common for machine learning algorithms.

Our response

We have now produced graphs in the supplementary material (S1 Figure) for the selection of hyperparameters including the activation function, learning rate, and the number of hidden nodes, and referenced from the methods section in the main text.

The pseudo-code for the imputation algorithm has now been added in the manuscript as a flow chart in Fig 2.

The original database collected from published articles with missing information, the complete database after filling the missing information by our neural network, and the neural network codes are made openly available at www.openaccess.cam.ac.uk. (ID: 6CB38B01-7F11-4041-A4F6-74F863C31946 and placeholder DOI link: https://doi.org/10.17863/CAM.52036). These links are now included in the manuscript, and moreover will also be disseminated in the webpage of the journal should the manuscript be accepted.

Reviewer comment

As mentioned above, the imputation function was alleged to be the highlight of current m.s. For validation of the effectiveness, I recommend the authors to conduct further computational experiment regarding imputation values. For instance, the authors may treat some known values as missing data in the cartilage data, and then evaluate the performance. I notice similar validation has been done in material data by some of the authors in reference [10], which can also be done here to strengthen the claim of current m.s..

Our response

We are grateful for the proposed computational experiment, which we have now included in the SI.

There is 18% data missing in the original dataset, which we can deliberately remove some data points randomly to make it more sparse and calculate the new R^2. The blue dots are the R^2 values from our neuron network models trained on a database with missing data ranging from 18% to almost 100%. The blue shaded area represents the uncertainty at each missing data percentage and the purple line is the fitted average result. We can observe the R^2 value smoothly decreased to 0 with a large missing data percentage, as the model is unable to explain the response with little information but to predict the average of the data.

This experiment further confirms the validation of our imputation functions.

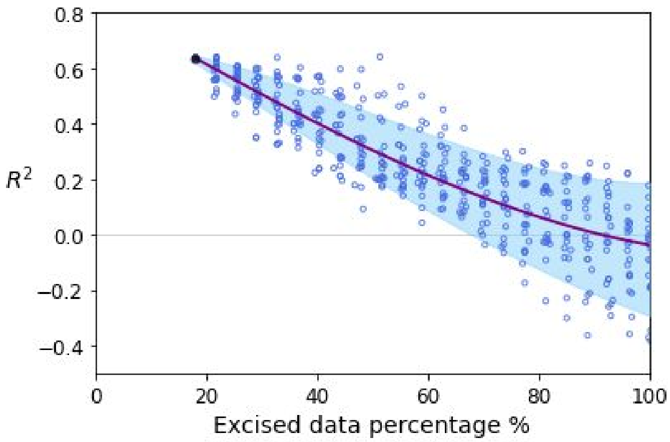

S2 Figure

We would also like to highlight the updated Fig 5B, which now demonstrates the performance of our model with the percentage of data imputed according to the uncertainty. The Fig 5B shows the predicted outcomes and the therapeutic outcomes from studies. If we impute the data by discarding the predicted values with large uncertainties, we will be more confident in making fewer predictions but with higher accuracy. This strategy is reflected in Fig 5 where the R^2 value increases with decreasing percentage of data imputed. 100% of data validated (the right-hand side of the graph) means we are predicting all of our data and calculating R^2 from it. 50% of data imputed means we only validate that data which have the 50% lowest uncertainty in their predictions.

Changes to the manuscript from line 463

The above plot was added in S2 Figure under the Supporting Information.

Reviewer comment

3. Comparison with other state-of-art machine learning methods.

The sample size of cartilage repair data used in this m.s. seems not to be overwhelmingly large, therefore neural network method might not out-perform other classical machine learning approaches by default. Since the m.s. used the word 'Machine Learning' instead of 'Neural

Network' in the article title, I suggest the authors to evaluate the performance of other powerful machine learning methods for continuous variables (e.g. random regression forests) to predict the outcome and compare the results (for instance, R-square in testing dataset) with neural network approach, hence highlighting neural network as the desired machine learning method and providing a baseline R-square for comparison.

Our response

We have now evaluated the performance of Random Forest (RF) on the trained database, and the R^2 value is 0.554 ± 0.006 based on the same validation method used for our neural network versus the R^2 value of 0.637 ± 0.005 for the method presented in the manuscript.
Note that the original experimental database has 18% missing entries (not available), most machine learning methods do not accept not available as an input and would simply drop those entries. We use our bespoke imputation algorithm to fill the missing data before supplying it to the Random Forest method. The RMSE results for RF with imputation and without imputation are now shown in S1 Figure(A). Our neural network method has an advantage over the RF method.

Other ML methods have also been conducted for comparison. The K-Nearest Neighbor (KNN) reported an optimal R^2 value of 0.336 ± 0.090 when we chose 3 nearest neighbors under Euclidean distance. Multiple Linear Regression (MLR) reported the R^2 value of -0.169 ± 0.120. The comparison graph using RMSE is now provided under S1 Figure(A).

The RMSE results for RF with imputation and without imputation are now shown in S1 Figure(A). It's difficult to have a fair comparison, but our neural network method clearly had an advantage over the random forest method.

We also implemented a Collective Matrix Factorization (CMF) model for comparison, the R^2 value is -0.003 ± 0.280. We think the poor performance might be due to the linearity assumption in the interaction of latent factors for CMF, which can be restrictive and fails to capture complex non-linear interactions.

Changes to the manuscript from line 165

We summarized the above in an entirely new subsection, "Other machine learning methods", comprised of the additional text:

We compare our neural network algorithm with a variety of other machine learning approaches in S1 Figure(A). Random Forest (RF) is a popular method, which builds an ensemble of decision trees to predict individual results. However, decision trees require all their input to be present during training that makes it impossible to build RF models using incomplete entries but to drop them, we use the imputation algorithm to fill the database and record the second-best R^2 value of 0.554 compared to the value of 0.637 from our neural network method. We have also tested the K-Nearest Neighbor (KNN) and Multiple Linear Regression (MLR) method, where 3 nearest neighbors were chosen as the optimal setting of KNN using Euclidean distance.

Another popular method of analyzing sparse databases is matrix factorization, where the matrix of condition and treatment values is approximately factorized into two lower-rank matrices that are then used to predict therapeutic outcomes for the new patient.
We used the modern Collective Matrix Factorization (CMF) implementation for comparison, and the hyperparameter alpha for the CMF model was chosen heuristically as 0.99. The R^2 value is -0.003, the reason might be the CMF method assumes linearity in the interaction of latent factors which fails to capture some complex non-linear interactions.

We also use the leave-one-out cross-validation to determine other hyperparameters of the neural network in S1 Figure.

Reviewer comment

1. On Line 40, it stated that 'We adapt a deep learning method ', which seems inaccurate. In fact, the authors only used very simple single-layer neural network in the current m.s., far from the widely perceived 'deep learning' that has multi-layer structure and huge amount of parameters.

Our response

We thank the Reviewer for pointing out the imprecise description. We rephrase the sentence to 'We adapt a neural network formalism'.

Reviewer comment

2. On line 91, the expression might be f(x)=y instead of f(x)=x.

Our response

We thank the Reviewer for bringing this confusing sentence to our attention. The sentence "We intended to find a function f that satisfies f(x) ≡ x for all entries in the database. " was rephrased to "We intended to find a function f that satisfies the fixed-point equation f(x) ≡ x for all entries in the database.”.

The trivial solution to the fixed-point equation is the identity operator, so that f(x) = x. However, this solution does not allow us to use the function f to impute data, and so we seek a solution to the fixed-point equation that by construction is orthogonal to the identity operator. This will allow the function to predict a given component of x from some or all other components.

Changes to the manuscript from line 90

We intended to find a function f that satisfies the fixed-point equation f(x) ≡ x for all entries in the database. The trivial solution to this fixed-point equation is the identity operator, f(x) = x, but this

solution does not allow us to impute data using the function f. We search for a solution to the fixed-point equation that is orthogonal to the identity operator, and allow the function to predict a given component of x from some or all other components.

Summary

We thank the Reviewer again for their conscientious review, which has helped us to improve the manuscript. We hope that we have addressed all of the questions of the Reviewer, meaning that the manuscript is now suitable for publication.

Response to Second Reviewer

We are grateful to the Reviewer for taking the time to carefully assess our work, and for the constructive feedback provided. We are pleased that the Reviewer found the topic interesting, and we address the Reviewer's comments in full below:

Reviewer comment

1. Methods: not enough detail, need to provide more details (some could be in supplement), e.g. why was this network topology chosen, number of hidden nodes. "adapt an EM algorithm" - details of this?

Our response

We thank the Reviewer for these helpful suggestions, we agree these are really important facts, which is also brought up by Reviewer 1. We repeat what we have done to the response to Reviewer 1.

Figures on the performance across different topologies or hyperparameters (learning rate, activation function, hidden nodes) are now provided in the Supplementary Information under S1 Figure. For the "EM algorithm", we have rewrite the paragraph. We also produced a flow chart (Fig. 2) to explain the procedure.

Changes to the manuscript from line 135.

We treated all properties as both inputs and outputs of the model and adapt an expectation-maximization algorithm. This is an iterative approach, where we first provided an estimate for the missing data and use the neural network to iteratively improve the initial estimate.

The algorithm is shown in Fig. 2.

For any unknown properties, we first set missing values to the average of the values present in the data set. With estimates for all values of the neural network we then recursively apply the following equation until convergence:

$$x^{n+1} = \gamma x^n + (1 - \gamma)\, x^n \,,$$

where n denotes the iteration step, x^n is a prediction for x obtained from the neural network. The converged result is then returned instead of $f(x^n)$. The function f remains fixed on each iteration of the cycle. A softening parameter, $\gamma \in [0, 1]$, is used to combine the results with the existing predictions, and a $\gamma > 0$ serves to prevent oscillations and divergences of the predictions. Typically, we set γ as 0.5.

Reviewer comment

2. Methods validation: It seems that this network topology successfully handles missing data by the leave-one-out training strategy. Some discussion of this strategy is needed. I.e. since the input data set is relatively small, would other imputation methods by (e.g.) matrix factorization, followed by a single hidden layer rather than several separate networks, work equally well or better? How does performance of this model compare to alternatives that may not handle missing data explicitly but could do so implicitly (e.g. a variational autoencoder)?

Our response

We have now evaluated the performance of Random Forest (RF) on the trained database, and the R^2 value is 0.554 ± 0.006 based on the same validation method used for our neural network versus the R^2 value of 0.637 ± 0.005 for the method presented in the manuscript.
Note that the original experimental database has 18% missing entries (NA), most machine learning methods do not accept NA as inputs and would simply drop those entries. We use the imputation algorithm to fill the missing data before supplying it to the Random Forest method. The RMSE results for RF with imputation and without imputation are now shown in S1 Figure(A). It's difficult to have a fair comparison, but our neural network method clearly had an advantage over the random forest method.

We also implemented a Collective Matrix Factorization (CMF) model for comparison, the R^2 value is -0.003 ± 0.280. We think the poor performance might be due to the linearity assumption in the interaction of latent factors for CMF, which can be restrictive and fails to capture complex non-linear interactions.

Other ML methods have also been conducted for comparison. The K-Nearest Neighbor (KNN) reported an optimal R^2 value of 0.336 ± 0.090 when we chose 3 nearest neighbors under Euclidean distance. Multiple Linear Regression (MLR) reported the R^2 value of -0.169 ± 0.120. The comparison graph using RMSE is now provided under S1 Figure(A).

Changes to the manuscript from line 165

We summarized the above in an entirely new subsection, "Other machine learning methods", comprised of the additional text:
We compare our neural network algorithm with a variety of other machine learning approaches in S1 Figure(A). Random Forest (RF) is a popular method, which builds an ensemble of decision trees to predict individual results. However, decision trees require all their input to be present during training that makes it impossible to build RF models using incomplete entries but to drop them, we use the imputation algorithm to fill the database and record the second-best R^2 value of 0.554 compared to the value of 0.637 from our neural network method. We have also tested the K-Nearest Neighbor (KNN) and Multiple Linear Regression (MLR) method, where 3 nearest neighbors were chosen as the optimal setting of KNN using Euclidean distance.

Another popular method of analyzing sparse databases is matrix factorization, where the matrix of condition and treatment values is approximately factorized into two lower-rank matrices that are then used to predict therapeutic outcomes for the new patient.
We used the modern Collective Matrix Factorization (CMF) implementation for comparison, and the hyperparameter alpha for the CMF model was chosen heuristically as 0.99. The R^2 value is -0.003, the reason might be the CMF method assumes linearity in the interaction of latent factors which fails to capture some complex non-linear interactions.

We also use the leave-one-out cross-validation to determine other hyperparameters of the neural network in S1 Figure.

Reviewer comment

3. Lack of reproducibility: in addition to the lack of written details on methodology, no source code nor any of the data used to train the model is provided. This is recognized as a growing concern in ML for biomedical studies (see https://arxiv.org/abs/2003.00898). For this study to be helpful to others, and to follow PLOS guidelines, working code should be provided along with documentation such that it can be followed by others. Data used as input could also be summarized in a supplement.

Our response

We have now made the original database from published studies, the pseudo source code for the imputation algorithm as Fig 2, the complete database with missing information filled by our neural network, and the source code of the neural network model available for further testing and validation.

The data and codes are available at www.openaccess.cam.ac.uk. (ID: 6CB38B01-7F11-4041-A4F6-74F863C31946 and placeholder DOI link: https://doi.org/10.17863/CAM.52036). These links are now included in the manuscript, and moreover will also be disseminated in the webpage of the journal should the manuscript be accepted.

Reviewer comment

4. Seven features are reported as provided best predictions, but the difference between 7 and 4 features seems to be very small. What is the R^2 for 4 features? Along with its variance via cross-validation? Are 4 predictors worse than 7?

Our response

We thank the Reviewer for bringing this inquiry to our attention. Yes, the difference is small. We did careful validation for different features, and the R^2 for 7 features is 0.637 ± 0.005, which is better than the R^2 for 4 features, 0.625 ± 0.012. The uncertainty here is based on standard deviation instead of variance via cross-validation. We agree the difference is not enormous, but

7 predictors have a higher R^2 value, which is one standard deviation away from the 4 predictors and have a smaller uncertainty in R^2 value, denoting it's the more robust. Further, 7 descriptors give better uncertainty prediction, which we will be using for imputation in Results.

It was also motivating those individual properties up to 7 predictors contribute to the R^2 value, and if we fit a parabola as a trend curve for the R^2 values (red line), we will also get the maximum at 7 descriptors. We updated the Figure 3 on page 7 with uncertainty plotted for each R^2 value and a horizontal line with R^2 = 0.637 for visual comparison.

Changes to the manuscript from line 214.

The top four properties give an R^2 value of 0.625 ± 0.012, and the combination of all seven positive properties has a maximum R^2 of 0.637 ± 0.005.
Overfitting was observed at a decreasing R^2 with more than seven descriptors, this happened when the system matched the training dataset but failed with unseen data draw from the validation dataset. Fewer descriptors did not provide a sufficient basis set, so we chose the first seven descriptors where each of them individually yields a positive R^2 value, which captured more correlations of clinical properties without overfitting and provided higher quality uncertainty prediction.

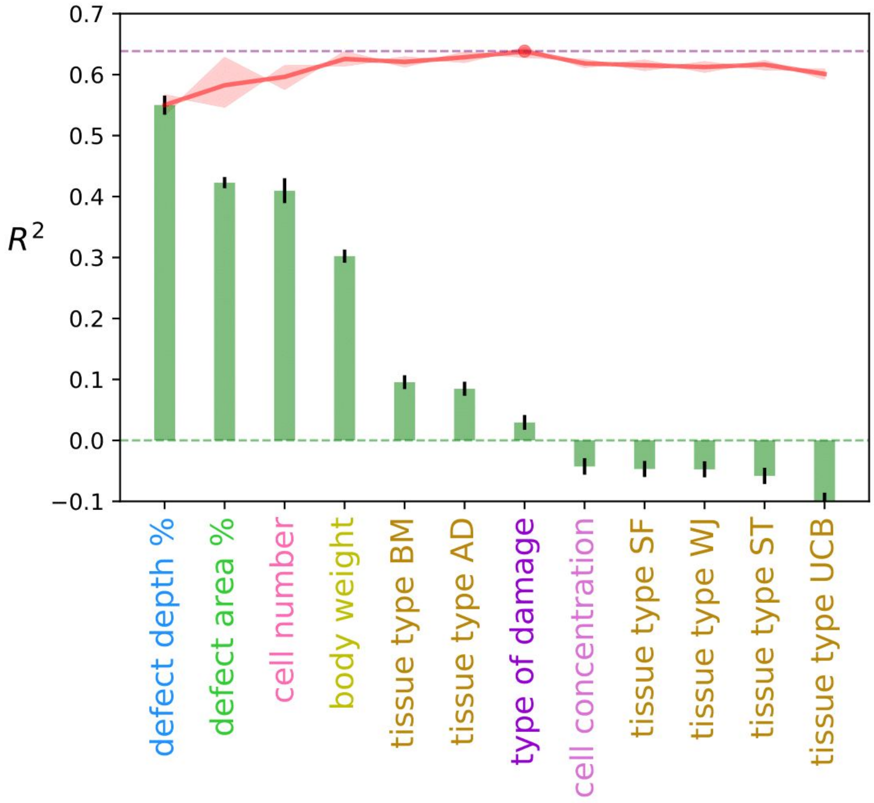

Figure 3

Reviewer comment

5. How closely correlated are the defect depth and defect area? And what possible effects may this have on predictions?

Our response

Defect depth and defect area are uncorrelated: we checked the Pearson's Correlation coefficient between the defect depth and the defect area, which is -0.238. Moreover, we checked the strength of correlation with another measure, Spearman's Rank Correlation, which is -0.067.

Another pair of descriptors is the defect depth percentage and defect area percentage, which takes consideration of the healthy cartilage dimensions of different species. The Pearson's Correlation coefficient between the defect depth percentage and the defect area percentage is 0.148, again showing that these two quantities are uncorrelated. Biologically, cartilage damaged by direct trauma has random defect geometries. In instances of chronic cartilage damage due to wear & tear or arthritis, there may be a weak correlation between the area & depth of the defect. However, the defect geometry can be complicated by the defect position, joint motion, and disease progression of individual patients, which would further weaken the correlation.

If two descriptors are correlated, it would be redundant to keep both and there is a possibility of overfitting. If there had been overfitting, this would have shown up in our cross-validation testing.

Following the Reviewer's important query further we also calculated the correlation information for all the descriptors and found no pairs of input descriptors have a coefficient larger than 0.53 using either Pearson's Correlation or Spearman's Rank Correlation coefficient.

Changes to the manuscript from line 206

A correlation test is performed between all properties to make sure no pairs of input properties are closely correlated, finding that both Pearson's Correlation and Spearman's Rank Correlation coefficients are smaller than 0.53.

Reviewer comment

6. More explanation of Fig. 4 is needed. It is not at all clear what is being presented here? RMSE zero without imputation and increases with imputation?

Our response

We thank the Reviewer for bringing this misleading figure to our attention. The x-axis label "missing data imputed %" was badly chosen and we replaced that with "Percentage of data

validated". 100% of data validated means we predicted and validated against every entry in our database, and all of these values contribute to the final R^2. 75% of data validated means that we calculate the R^2 using only the 75% of the data sorted by the uncertainty in their predictions.

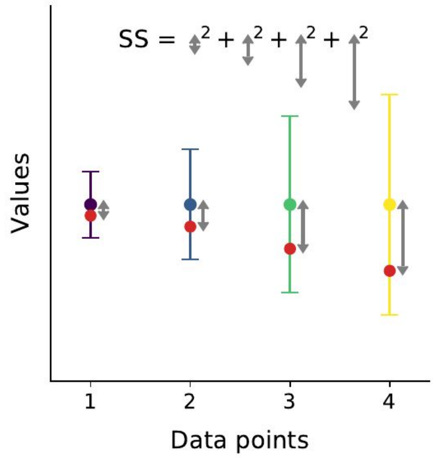

Figure 5A

The Figure above shows an example when making predictions for just four data points. The colored dots represent the predicted value and their uncertainty that is also predicted by the machine learning method is shown by the colored bars (magenta, turquoise, green, and yellow), the red dots are the true (unknown to machine learning) values used for validation, and the difference between predicted and true values are measured as the grey arrows. The sum of square (SS) errors is then used to calculate R^2. When we disregard those with larger uncertainty in prediction (grey bar), there should be a high likelihood of discarding data points with large error.

Moreover, we have updated the Figure 5A as shown above so that the colors of the bars better align with the colors shown in Figure 5B, which we feel further improves the clarity of the presentation of this important concept.

The R^2 value using that fraction of data validated is plotted in Fig 5B demonstrates a higher accuracy when we impute the data by discarding the predicted values with larger uncertainties, if our uncertainty predictions had been poor then the R^2 would have increased. It is again color-coded by uncertainty ranking. The best R^2 value of 0.743 was reported at 82% of data being validated, and then reached the plateau when less than 70% of data are being validated. Validating fewer data can lead to significant noise and is less applicable in the real-world where we wish to impute as much as possible, therefore we focus on the >50% regime. We have now revised the figure and hope that we have demonstrated a higher accuracy can be achieved with imputation.

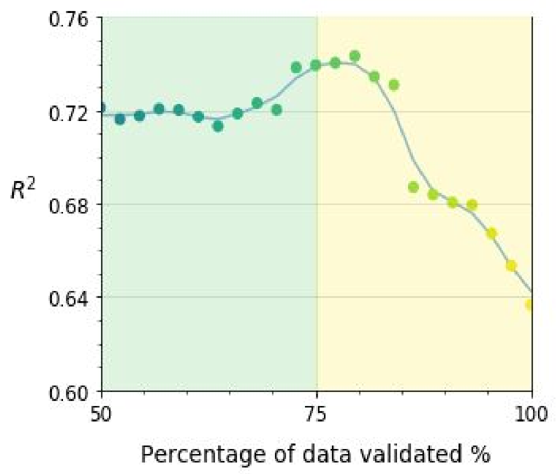

Figure 5B

Reviewer comment

7. In Fig. 6, sharp drop offs are observed for both parameters. Is this reflecting a real biological prediction or is it due to lack of enough data to “fill-in” - I.e. make more continuous - the landscape in fig 6?

Our response

The study showed that critical thresholds of damage exist for effective tissue repair to happen, which is similar in the case of volumetric muscle loss [55]. In cartilage repair models, a "critical size" osteochondral defect that cannot effectively repair by itself, has been widely used. In most cases, such critical sizes were applied at estimated default values for different animal models. Some studies have attempted to experimentally determine the critical size of the defect in terms of depth and diameter in specific animal models [56]. In our machine learning model, we predicted more accurate "critical size" defects in human in terms of the percentage of each individual's cartilage depth and area. Furthermore, our model identified multiple sharp drop-offs, indicating the presence of multiple "critical sizes" that limit the cartilage repair to different levels of post MSC therapy. As a result, we believe that our machine learning provides more insights in quantitative determination of patient- and therapy-specific critical size cartilage defects, which would serve as an important reference for orthopaedic clinicians to design MSC therapy.

Changes to the manuscript from line 336

The study showed that critical thresholds of damage exist for effective tissue repair to happen, which is similar in the case of volumetric muscle loss [55]. In cartilage repair models, a “critical size” osteochondral defect that can not effectively repair by itself, has been widely used. In most cases, such critical sizes were applied at estimated default values for different animal models. Some studies have attempted to experimentally determine the critical size of the defect in terms of depth and diameter in specific animal models [56]. In our machine learning model, we predicted those “critical size” defects in human as we observed a rapid decrease in the normalized cartilage repair score when the defect area percentage increases from 6% to 35%.

Another fast drop was observed at 64%, and minimal repair should be expected when more than 70% of cartilage area is damaged. Those sharp drop-offs identified from the model indicates the presence of multiple “critical sizes” that limit the cartilage repair to different levels of post-MSC therapy. These quantitative cartilage repair predictions based on the patients' defect conditions provide useful references for the clinicians to make decisions on the therapy. The actual therapeutic outcome of these patients should be used to further train the model to improve the predictive power.

Added references [55] & [56]

[55] Anderson SE, Han WM, Srinivasa V, Mohiuddin M, Ruehle MA, Moon JY, et al. Determination of a critical size threshold for volumetric muscle loss in the mouse quadriceps. Tissue Engineering Part C: Methods. 2019; 25(2):59-70.

[56] Gotterbarm T, Breusch S, Schneider U, Jung M. The minipig model for experimental chondral and osteochondral defect repair in tissue engineering: retrospective analysis of 180 defects. Laboratory animals. 2008; 42(1):71-82.

Reviewer comment

Minor comments

- Fig. 5: 3D pie charts are not good ways of representing data as they skew the actual pie chart! (“closer” slices look bigger) Please choose alternative method for visualizing. 2D pie charts are ok.

Our response

We thank the Reviewer for pointing out the distort effect of the 3D pie chart. We have now followed the reviewer’s suggestion and updated the figure to a 2D pie chart.

Reviewer comment

- Fig. 8 roadmap - this figure does not seem pertinent to the work done in the paper. Does not contain info relevant to the work done. Update or remove.

Our response

We have now removed the Fig.8 roadmap.

Reviewer comment

- Please edit thoroughly and carefully for English language errors, eg plural of “mechanism” incorrect in multiple places,

- Line 267 typo “we now studying”

Our response

We thank the Reviewer for pointing out the typographical errors, we have carefully proof-read the manuscript, and have corrected the following typographical errors:

- Abstract, a MSC therapy -> an MSC therapy
- Author Summary, life qualify -> life quality
- Author Summary, complex mechanism -> complex mechanisms
- Author Summary, provides important reference -> provides an important reference
- Introduction, Line 16, cellular mechanism -> cellular mechanisms
- Introduction, Line 27, requires quantitative assessment -> requires a quantitative assessment
- Introduction, Line 30, To overcome these shortcomings -> To overcome these shortcomings,
- Methods, Line 87, as follow, -> as follows,
- Methods, Line 121, by neural network -> by a neural network
- Methods, Line 169, are negative correlated -> are negatively correlated
- Results, Line 225, from neural network -> from the neural network
- Results, Line 267, we now studying -> we now study
- Results, Line 279, Similarly -> Similarly,
- Results, Line 316, and high dose -> and a high dose
- Results, Line 340, Although fundamental difference -> Although a fundamental difference
- Results, Line 341, the mechanism of cartilage repair -> the mechanisms of cartilage repair
- Discussion, Line 363, The assessment of new patient -> The assessment of new patients

(The line number in this response is based on the first submitted version.)

Summary

We thank the Reviewer again for their detailed and constructive comments, which have motivated us to improve many aspects of our work. We hope that all the questions are properly addressed, and the manuscript is now ready for publication in the PLOS Computational Biology.
