# Supplementary material for: Machine learning to predict mesenchymal stem cell efficacy for cartilage repair
Source: PLoS Comput Biol. 2020 Oct 7;16(10):e1008275. doi: 10.1371/journal.pcbi.1008275 (PMC7571701; doi:10.1371/journal.pcbi.1008275)
Supplement: Attachment — Submitted filename: 2nd_response_letter.pdf [file pcbi.1008275.s003.pdf]

Manuscript No.: PCOMPBIOL-D-20-00130R1

Title: Machine learning to predict mesenchymal stem cell efficacy for cartilage repair

Dear Editor,

We are delighted to know that the Reviewers found the manuscript much improved and we thank the Reviewers for their helpful comments. We have addressed the confusions and updated the manuscript following their suggestions in full below. We hope that following these changes the manuscript is now ready for publication in the PLOS Computational Biology.

Yours faithfully,

Yu Yang Fredrik Liu, Dr Yin Lu, Professor Steve Oh, and Dr Gareth Conduit

Response to First Reviewer

We are grateful to the Reviewer for taking the time to carefully review our revised manuscript and providing us with constructive feedback again. We are pleased to hear that the Reviewer found the manuscript improved on method details and that we have addressed the major concerns. We now address the Reviewer's remaining comments and suggestions in full below:

Reviewer comment 1

In the clean version of m.s. line 69-71, it is said that the input properties are the various factors, and output properties are outcomes measured by scores. However, in line 134, it states all properties are both inputs and outputs of the models. I suggest the authors to be more specific and explicit here, eliminating the inconsistency, since general readers are very likely to have the same confusion with me.

I recommend to clarify the exact meanings of the variables in Figure 1, in the concrete text of cartilage repair data – for example, the vector x simply represents various factors in data, or both factors and outcomes? And the authors may explain in detail how to predict outcomes when using only the factors given a new test data? Also, when computing R-square, what variables are used?

Our response

We thank the Reviewer for bringing this confusing sentence to our attention. The treatment conditions (input) and therapeutic outcome (output) are meant to be specific for the cartilage repair treatment. We replaced the sentence “We treated all properties as both inputs and outputs of the model and adapt an expectation-maximization algorithm” in line 134 with “A typical neural network formalism requires each property to be either an input or output of the

network, and all inputs must be provided to compute a valid output. In contrast, our neural network takes the known treatment conditions and the therapeutic outcome (if known) as inputs then outputs the predictions for unknown treatment conditions and the therapeutic outcome. Then following the flowchart in Fig 2 the neural network is applied iteratively to cycle the predictions of the unknown treatment conditions and therapeutic outcome until self-consistency, an expectation-maximization algorithm." In the special but important case of known treatment conditions but unknown therapeutic outcome, the neural network will input the treatment conditions and run just once to predict the unknown therapeutic outcome.

We have also clarified the variables in Figure 1, with specific examples of variables at the following lines in the clean version:

Line 88, Each entry $x = (x_1, ..., x_I)$ to the neural network is a vector of length I, with the first $I-1$ variables being the distinct treatment conditions (including species, body weight, tissue source, cell number, cell concentration, defect area, defect depth, and type of cartilage damage); and the final Ith variable is the therapeutic outcome.

Line 97, The output $(y_1, ..., y_I)$ is a vector of length I, with the first $I-1$ variables being the predicted treatment conditions (if unknown); and the final Ith variable is the therapeutic outcome.

Line 107, There are exactly as many given properties as predicted properties, since all types of properties are treated equally by the ANN.

Line 161, In this work, we are only using the therapeutic outcome as the variable for R^2.

Line 163, where y_i^{actl} is therapeutic outcome from the ith case (patient or animal) and y_i^{pred} is the corresponding prediction.

Reviewer comment 2

More details about variable selections in benchmarking

When comparing with other methods, the m.s. seems not state how the features are selected optimally for linear regression or KNN. From Methods and Figure 3, we know variable selection is important to improve the R-square of authors' model. For sake of fair benchmarking, I suggest the authors may also tune the parameters or especially do feature selections for other machine learning algorithms. Also, the RF algorithm can run with incomplete data using some naïve imputation strategies, instead of author's approach, which might also be incorporated into comparison. These details should be clearly recorded in the manuscript or SI to improve the reproducibility and soundness of the paper.

Our response

We thank the Review for the helpful suggestion. In supplementary information S1 Figure, we used the same feature selected from our neural network in other machine learning methods while tuning other hyperparameters. Following the Reviewer's suggestion, we have now performed feature selection validation and plotted additional figures similar to Figure 3 here. Improvements have been seen in all three methods, and RF remains a strong candidate next to NN.

We have updated the S1 Fig(A) and the following lines in the supplementary information:

> Line 442, In S1 Fig(A) we compare to other machine learning algorithms, Neural Network (NN), Random Forest (RF), K-Nearest Neighbor (KNN), Collective Matrix Factorization (CMF), and Multiple Linear Regression (MLR). For each method, we also perform individual investigation on the selection of input properties and optimize its hyperparameters to deliver maximum accuracy and measure through the same leave-one-out cross-validation test as in the paper. Note the CMF algorithm has an internal component selection parameter so we did not manually select the input properties for it. For RF, KNN, and MLR that require all inputs to be present, we either use imputation algorithm to fill in the database or perform machine learning using only complete rows of data, and also measure accuracy using machine learning model trained on now complete data.

For the Random forest (RF) algorithm, we thank the Reviewer for pointing out other imputation strategies are available and making comparisons between different imputation strategies is certainly an interesting topic. To implement the same strategy for other machine learning methods for benchmarking may be beyond the current scope of the manuscript, but we will keep that in our mind for the future exploration.

Additional information for the feature selection.

Feature selection for Multiple Linear Regression (MLR).

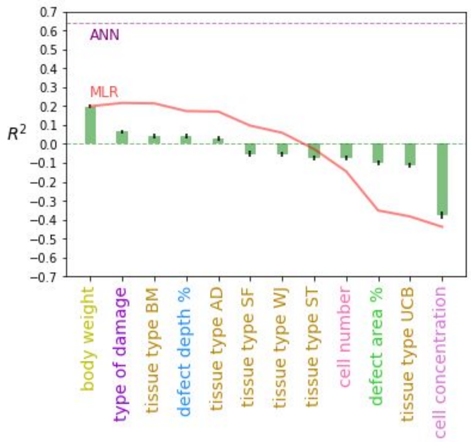

Feature selection for K-nearest Neighbour (KNN).

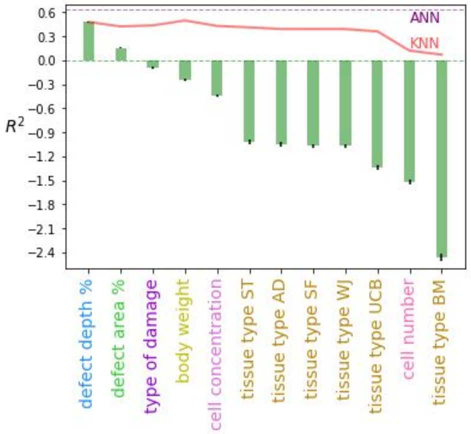

Feature selection for Random Forest (RF).

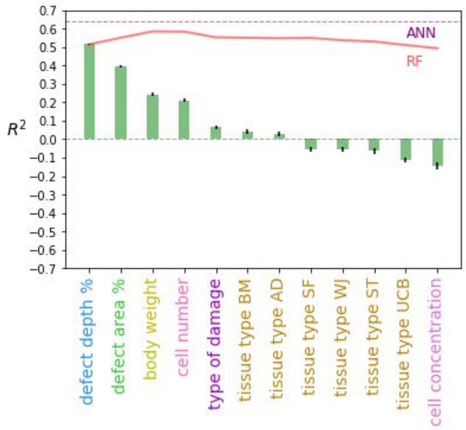

Reviewer comment 3

Accessibility of data/codes
By the time of submitting the review report, I still cannot get access to the data and codes using provided webpage or placeholder doi. It shows the doi not found, and I cannot find where to enter the ID on https://www.openaccess.cam.ac.uk/. I will be grateful if the authors can provide me more instructions to find the data, or the direct link to the database.

Our response

We apologize for the access issue with the *openaccess.cam.ac.uk* platform. We asked the university team, and were informed the database will become publicly available should the manuscript be accepted and no changes can be made, the placeholder DOI will also be activated after they checked the metadata and data files. But the *openaccess* platform is unable to share links for reviewing at this stage. We indicated that the URLs/DOIs will be available only after acceptance under the additional data availability information during submission.

For the interim we provide a link to directly access the database from the group website,
http://www.tcm.phy.cam.ac.uk/~ly297/PLOS_Compute_Biol_Database.tar.gz
or from the link provided at the bottom of http://www.tcm.phy.cam.ac.uk/profiles/ly297/.

Summary

We thank the Reviewer again for their conscientious review, which has helped us to improve the manuscript. We hope that we have addressed all of the questions of the Reviewer, and the manuscript is now suitable for publication.

Response to Second Reviewer

We are grateful to the Reviewer for taking the time to reassess our work. We are pleased that the Reviewer found most points in the previous review have been clarified, and we address the Reviewer's concerns in full below:

Reviewer comment 1

Fig 5A is not at all clear, much more detail needed, e.g. "Values" and "Data points" and not meaningful axis labels. Is the point that the data points are ordered by uncertainty? Perhaps mark specifically where each point in 5A comes from in 5B? It is also not clear what the relationship is between the uncertainty in estimates and the error (distance between true value and predicted) and what the significance of this is?

Our response

We thank the Reviewer for bringing this figure to our attention, the figure demonstrates the confidence in predictions that can be used to improve the accuracy of results. We have clarified 5A, putting a definitive y-axis scale of therapeutic outcome whose data points are selected from 5B, ordered by their uncertainty. The x-axis simply delineates four different sample predictions. In practice, to calculate the R^2 value (a single point) in Fig 5B, all the data points (not shown explicitly in Fig 5A) will be used to obtain the sum of squares first then scaled to obtain the R^2 value. Those predictions with large uncertainties (colored bars, e.g. the yellow bar) have a higher likelihood with large errors (grey arrows). If we discard those with large uncertainties from our prediction, we will have a new updated R^2 value. The amount of data imputed or validated vs their R^2 value is shown in Fig 5B.

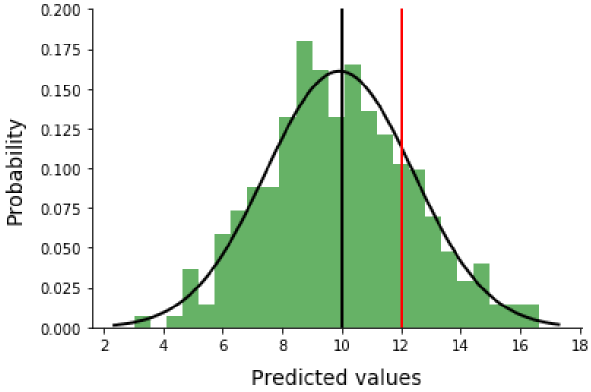

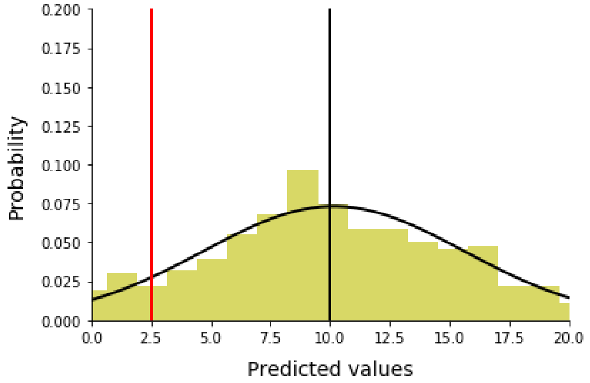

For the relationship between the uncertainty in estimates and the error, if we are able to understand the uncertainty in our prediction, we can then drill down to focus on the high quality predictions. When our neural network is making predictions for one data point, it generates the

probability distribution of the results as well as the predicted value. There are two distinct cases, a narrow or broad distribution:

Narrow: Sometimes the probability distribution will look like the one on the left, which has a sharp and narrow peak around the mean value, so the neural network is confident in that prediction. The real answer (red line) should be close to the mean prediction (black vertical line).

Broad: On the other hand, we have situations that look like the one on the right, where we have a broad, flat probability distribution. The neural network lacks confidence in the prediction, reflected in the large error, and therefore the real answer (red line) might be somewhere far away from the mean prediction (e.g. in the left corner of the plot).

The distinction between these distributions is useful when making decisions for the predicted values. We should discard predictions with a broad probability distribution (such as the illustration on the right) and instead just focus on predictions where we can be confident about the result, such as on the left. By doing this, we make fewer predictions overall but we can be confident that they are of higher quantity and therefore more useful in practice.

Changes to the manuscript from line 241.

In particular, we can discard predictions carrying large uncertainty, and trust only those with smaller uncertainty. The idea is illustrated in Fig 5A, where we select four of the points from 5B, including that with the largest uncertainty that has the highest likelihood of deviating from the true value so should give the largest error, as well as other quartiles in uncertainty. This allowed us to focus on the most confident predictions only at the expense of reporting fewer predictions, e.g. discard the data point with the largest uncertainty (yellow bar) and recalculate the sum of squares for the R^2 value. By doing so, the quality of the remaining neural network predictions increases as the root-mean-square error between the predicted values and the actual values decreases when a smaller fraction of predictions is accepted and validated as shown in Fig 5B.

We have also replotted the Fig 5A.

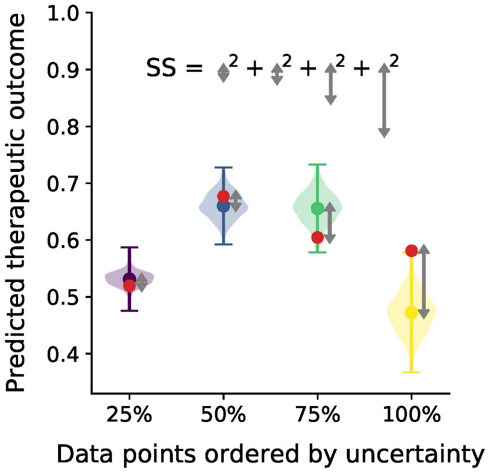

We updated the label for x-axis and y-axis to *data points ordered by uncertainty* and *predicted therapeutic outcome* respectively. We also updated the x-axis ticks to 25%, 50%, 75%, 100% instead of 1, 2, 3, 4 and the data points are ordered by their uncertainty, which also corresponds to the percentage of data validated in Fig 5B. The violin represents the probability density distribution of the predicted results, which we hope improves the clarity of the presentation of this concept.

Reviewer comment 2

"The data and codes are available at www.openaccess.cam.ac.uk . (ID: 6CB38B01-7F11-4041-A4F6-74F863C31946 and placeholder DOI link: https://doi.org/10.17863/CAM.52036" Neither the doi link nor the ID in openaccess.cam database work for me. Thus there is still no available code associated with this manuscript.

Our response

We apologize for the access issue with the *openaccess.cam.ac.uk* platform. An inquiry was sent to the university team, and we were informed that the database will become publicly available should the manuscript be accepted and no changes can be made, the placeholder DOI will also be activated after they checked the metadata and data files. But the *openaccess.cam.ac.uk* platform is unable to share links for reviewing at this stage. We indicated that the URLs/DOIs will be available only after acceptance under the additional data availability information during submission.

As an interim solution we provide a link to directly access the database from the group website, http://www.tcm.phy.cam.ac.uk/~ly297/PLOS_Compute_Biol_Database.tar.gz
or from the link provided at the bottom of http://www.tcm.phy.cam.ac.uk/profiles/ly297/.

Summary

We thank the Reviewer again for their constructive comments, which have motivated us to improve many aspects of our work. We hope that all the questions are properly addressed, and the manuscript is now ready for publication in the PLOS Computational Biology.
