# Supplementary material for: Machine learning to predict mesenchymal stem cell efficacy for cartilage repair
Source: PLoS Comput Biol. 2020 Oct 7;16(10):e1008275. doi: 10.1371/journal.pcbi.1008275 (PMC7571701; doi:10.1371/journal.pcbi.1008275)
Supplement: Attachment — Submitted filename: 3rd_response_letter.pdf [file pcbi.1008275.s004.pdf]

Manuscript No.: PCOMPBIOL-D-20-00130R1

Title: Machine learning to predict mesenchymal stem cell efficacy for cartilage repair

Dear Editor,

We are glad to know that the Reviewers found the manuscript has addressed all concerns regarding the scientific aspects and we thank the Reviewers for their comments. We have improved the writing of the manuscript and updated the supplementary information. We hope that following these changes the manuscript is now ready for publication in the PLOS Computational Biology. We would also like to emphasize the manuscript is submitted for a research article instead of a software submission.

Yours faithfully,

Yu Yang Fredrik Liu, Dr Yin Lu, Professor Steve Oh, and Dr Gareth Conduit

Response to First Reviewer

We are grateful to the Reviewer for taking the time to carefully review our revised manuscript and providing us with feedback again. We are pleased to hear that the Reviewer found the manuscript has addressed most concerns regarding the scientific aspects. We now address the Reviewer's remaining comments and suggestions in full below:

Reviewer comment 1

In the files provided by authors, I can only find a simple python file defining the class of Neural Network, which is quite routine and not novel. I cannot figure out how the data imputation (EM algorithm), claimed as the major contribution and novelty in current work, is achieved in the code. Nor do the authors provide script to reproduce their key findings in the manuscript using the cartilage repair database.

Note it is the policy of the PLoS CB that "authors must clearly provide detail, data, and software to ensure readers' ability to reproduce the models, methods, and results". The reproducibility issue is especially important in the field of machine learning. I therefore strongly recommend the authors to at least provide 1) the script to reproduce their key results in the manuscript using their dataset, 2) brief tutorials or documentations about their defined functions or class in a user-friendly way, making it convenient for the interested readers to directly implement the algorithm (especially data imputation and uncertainty quantification) in other datasets and do their desired benchmarking to validate the algorithms. Overall, I do think that open, reproducible scripts and clear documentations about the proposed algorithms are necessary before this manuscript is accepted.

Our response

We thank the Reviewer for emphasising the reproducibility issue in the field of machine learning.

To summarize for the previous revisions, we have provided the details for the imputation algorithm in the subsection *Handling missing data* under *Methods* and a flowchart in *Fig 2. Data imputation algorithm for the vector* ***x*** was added in the manuscript for illustration. The pseudo-code for uncertainty calculation was shown in *S1 Algorithm: A ensemble model to measure the ANN's prediction uncertainty.* The original database gathered from the literature, and a 'complete' database with missing information filled from our neural network are also included, along with a sample neural network architecture file in Python.

We provide a Python notebook comprising a neural network that delivers the performance and results described in the manuscript. Documentation in the form of comments and installation guide is included in the Python notebook, and we outline the main functionality here. The outline structure of the code is:

Section	Functionality
1	An implementation of a neural network (MLPRegressor) from the *Scikit-learn* library
2	Three imputation methods, including the capability to handle missing data. The first two implementations in Sec. 2a and Sec. 2b used the *Scikit-learn* library in Python, the third one in Sec. 2d is our implementation in the manuscript.
2c	Where the user can perform standard imputation. This implementation used the *Scikit-learn* library in Python.
2d	Where the user can perform our imputation and iteratively update the missing value as a function of the imputed value and the predicted output
3	Where the user can import the database
4	Where the user can tune the neural network parameters
5	The prediction of one entry along with the uncertainty of the prediction.
6a	Leave-one-out cross-validation method implemented in Bash, which can run the above neural network script for every entry in the dataset.
6b	An implementation of the leave-one-out cross validation when performing the calculation of the coefficient of determination, R^2, in Python
7	An implementation of the ensemble model by sampling the dataset in Python<br>Printing of the coefficient of determination, R^2

This Python notebook along with the methods described in the manuscript provides sufficient details for other interested readers to either extend this script or write their own scripts and reproduce the results in the paper.

Just to confirm, the *openaccess* platform is unable to share the data during this reviewing stage. For the interim we provide a link to directly access the database along with a spreadsheet with all numerical data that underlies graphs in the manuscript, http://www.tcm.phy.cam.ac.uk/~ly297/PLOS_Database.tar.gz .

Summary

We thank the Reviewer again for their suggestion. We hope that we have addressed all of the concerns of the Reviewer, and the manuscript is now suitable for publication.

Response to Second Reviewer

We are grateful to the Reviewer for taking the time to reassess our work. We are pleased that the Reviewer found that all the questions are properly addressed.
